# Spread of shingles impeded by previous surgical scar?

**DOI:** 10.1002/ski2.417

**Published:** 2024-06-24

**Authors:** Makoto Shiraishi, Masakazu Kurita

**Affiliations:** ^1^ Department of Plastic and Reconstructive Surgery The University of Tokyo Hospital Tokyo Japan

## Abstract

We herein report an atypical case of shingles caused by varicella zoster virus after surgery. The virus, which goes along with the cutaneous nerve from the ganglia, was unable to spread across the previous surgical scar due to damage of the cutaneous nerves, but made a detour to the upper left direction from the scar.

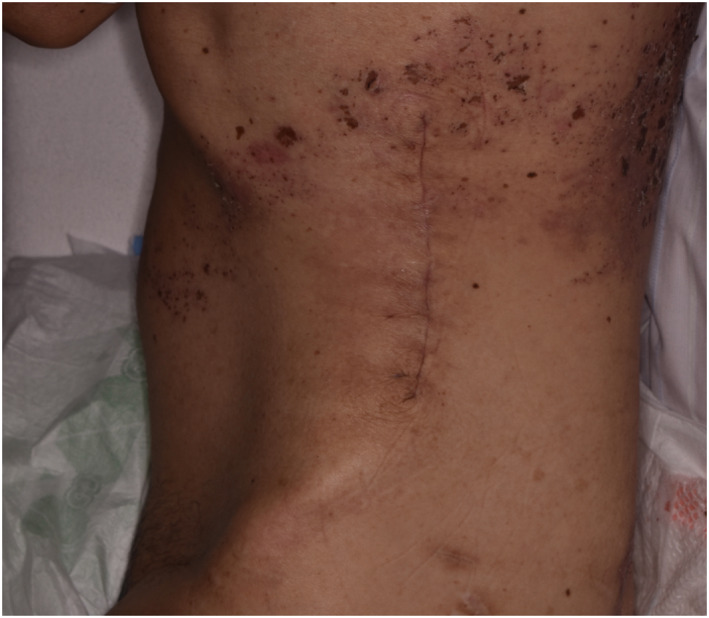

We present a case of a 76‐year‐old man diagnosed clinically with shingles which occurred after two surgical treatments for pressure ulcers. Upper‐body blisters appeared on the left lateral thoracic region 4 days after the second operation, which turned into scabs a week later. Around the surgical scar, these scabs were only seen on the dorsal side of the surgical scar at the first operation; however, the scabs above the scar level extended from the dorsal to the ventral side (Figure 1), indicating that varicella zoster virus was unable to spread across the scar due to damage to the cutaneous nerves.

**FIGURE 1:**
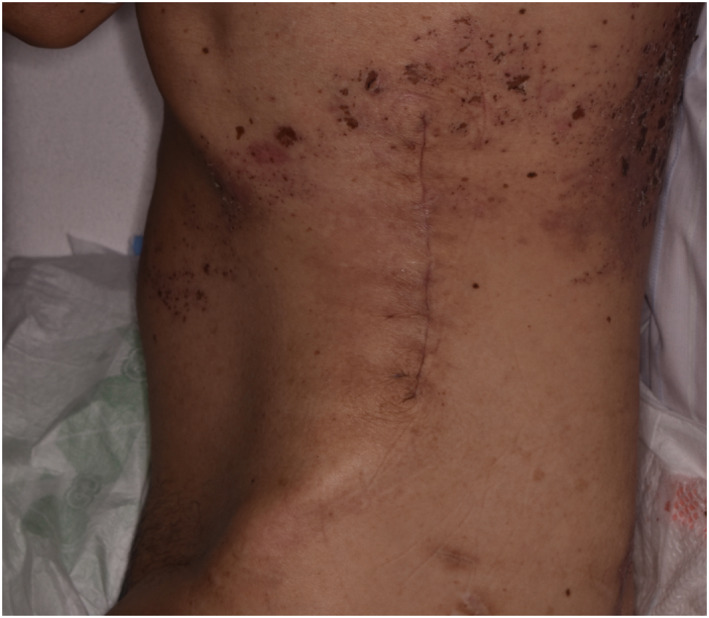
Scabs of herpes zoster around the previous surgical scar. The varicella‐zoster virus could not cross the surgical scar, but made a detour to the upper left direction from the scar.

## CONFLICT OF INTEREST STATEMENT

None to declare.

## AUTHOR CONTRIBUTIONS

**Makoto Shiraishi**: Conceptualization (lead); data curation (lead); formal analysis (lead); investigation (lead); methodology (lead); software (lead); validation (lead); visualization (lead); writing – original draft (lead); writing – review & editing (lead). **Masakazu Kurita**: Conceptualization (supporting); project administration (lead); supervision (lead); writing – review & editing (supporting).

## ETHICS STATEMENT

Not applicable.

## PATIENT CONSENT

Patient consent was obtained for publication.

## Data Availability

The article's underlying data will be shared on reasonable request to the corresponding author.

